# Supplementary material for: Description of a new Pangasius (Valenciennes, 1840) species, from the Cauvery River extends distribution range of the genus up to South Western Ghats in peninsular India
Source: PeerJ. 2022 Nov 8;10:e14258. doi: 10.7717/peerj.14258 (PMC9651045; doi:10.7717/peerj.14258)
Supplement: Supplemental Information 2 [file peerj-10-14258-s002.doc]

| **S. No.** | **Species** | **Accession Number** | **Location** |
| --- | --- | --- | --- |
|  | *Pangasius icari* sp. nov. | OK480013 | Mettur Dam, Tamil Nadu, River Cauvery |
|  | *Pangasius icari* sp. nov. | OK480014 | Mettur Dam, Tamil Nadu, River Cauvery |
|  | *Pangasius icari* sp. nov. | OK480046 | Mettur Dam, Tamil Nadu, River Cauvery |
|  | *Pangasius icari* sp. nov. | OK480047 | Mettur Dam, Tamil Nadu, River Cauvery |
|  | *Pangasius icari* sp. nov. | OK480048 | Mettur Dam, Tamil Nadu, River Cauvery |
|  | *Pangasius icari* sp. nov. | OK480049 | Mettur Dam, Tamil Nadu, River Cauvery |
|  | *Pangasius icari* sp. nov. | OK480050 | Mettur Dam, Tamil Nadu, River Cauvery |
|  | *Pangasius icari* sp. nov. | OK480051 | Mettur Dam, Tamil Nadu, River Cauvery |
|  | *Pangasius icari* sp. nov. | OK480052 | Mettur Dam, Tamil Nadu, River Cauvery |
|  | *Pangasius icari* sp. nov. | OK480053 | Mettur Dam, Tamil Nadu, River Cauvery |
|  | *Pangasius icari* sp. nov. | OK480054 | Mettur Dam, Tamil Nadu, River Cauvery |
|  | *Pangasius silasi* | KM232631 | Nagarjuna Sagar Dam, Andhra Pradesh, River Krishna |
|  | *Pangasius silasi* | KM232632 | Nagarjuna Sagar Dam, Andhra Pradesh, River Krishna |
|  | *Pangasius silasi* | KM232633 | Nagarjuna Sagar Dam, Andhra Pradesh, River Krishna |
|  | *Pangasius silasi* | KM232634 | Nagarjuna Sagar Dam, Andhra Pradesh, River Krishna |
|  | *Pangasius silasi* | KM232635 | Nagarjuna Sagar Dam, Andhra Pradesh, River Krishna |
|  | *Pangasius silasi* | KM434888 | Nagarjuna Sagar Dam, Andhra Pradesh, River Krishna |
|  | *Pangasius silasi* | KM434887 | Nagarjuna Sagar Dam, Andhra Pradesh, River Krishna |
|  | *Pangasius silasi* | KM434889 | Nagarjuna Sagar Dam, Andhra Pradesh, River Krishna |
|  | *Pangasius pangasius* | JX997836 | - |
|  | *Pangasius pangasius* | EU871046 | - |
|  | *Pangasius pangasius* | EU871047 | - |
|  | *Pangasius pangasius* | EU871048 | - |
|  | *Pangasius pangasius* | KC572135 | - |
|  | *Pangasius pangasius* | KM232618 | Bejjur, Telangana, River Pranahita |
|  | *Pangasius pangasius* | KM232620 | Bejjur, Telangana, River Pranahita |
|  | *Pangasius pangasius* | KM232621 | Bejjur, Telangana, River Pranahita |
|  | *Pangasius pangasius* | KM232622 | Beldanga, West Bengal, River Bhagirathi |
|  | *Pangasius pangasius* | KM232623 | Farakka Barrage, West Bengal, River Ganga |
|  | *Pangasius pangasius* | KM232624 | Farakka Barrage, West Bengal, River Ganga |
|  | *Pangasius pangasius* | KM232625 | Farakka Barrage, West Bengal, River Ganga |
|  | *Pangasius pangasius* | KM232626 | Naraj Barrage, Odisha, River Mahanadi |
|  | *Pangasius pangasius* | KM232627 | Jobra Barrage, Odisha, River Mahanadi |
|  | *Pangasius pangasius* | KM232628 | Jobra Barrage, Odisha, River Mahanadi |
|  | *Pangasius pangasius* | KM232629 | Jobra Barrage, Odisha, River Mahanadi |
|  | *Pangasius pangasius* | KM232630 | Dompara, Assam, River Brahmaputra |
|  | *Pangasius mekongensis* | KT289880 | Vietnam |
|  | *Pangasius larnaudii* | EU752152 | - |
|  | *Pangasius nasutus* | JF781172 | - |
|  | *Pangasius nasutus* | JF781173 | - |
|  | *Pangasius nasutus* | JF781174 | - |
|  | *Pangasius nasutus* | JF781175 | - |
|  | *Pangasius sanitwongsei* | KC627282 | - |
|  | *Pangasius sanitwongsei* | KC627283 | - |
|  | *Pangasius bocourti* | EF609425 | - |
|  | *Pangasius bocourti* | JF292427 | - |
|  | *Pangasius bocourti* | JF292428 | - |
|  | *Pangasius bocourti* | JF292429 | - |
|  | *Pangasius krempfi* | KT289877 | - |
|  | *Pangasianodon hypophthalmus* | GU324201 | - |
|  | *Pangasianodon hypophthalmus* | KM232616 | Bejjur, Telangana, River Pranahita |
|  | *Pangasianodon hypophthalmus* | KM232617 | Bejjur, Telangana, River Pranahita |
|  | *Pangasianodon hypophthalmus* | EF609427 | - |
|  | *Horabagrus brachysoma* | JX460967 | - |
|  | *Clupisoma garua* | JN628921 | - |
|  | *Catla catla* | JX983237 | Hoshangabad, Madhya Pradesh, River Narmada |
|  | *Xenomystus nigri* | NC012715 | - |
|  | *Chitala chitala* | KX894524 | - |
