# Supplementary material for: Description of a new Pangasius (Valenciennes, 1840) species, from the Cauvery River extends distribution range of the genus up to South Western Ghats in peninsular India
Source: PeerJ. 2022 Nov 8;10:e14258. doi: 10.7717/peerj.14258 (PMC9651045; doi:10.7717/peerj.14258)
Supplement: Supplemental Information 3 [file peerj-10-14258-s003.doc]

10_Pangasius_icaria_sp_nov.

CAGCCCTTAGCCTGCTAATTCGGGCTGAGCTAGCCCAGCCCGGCGCCCTTCTGGGTGATG

ACCAAATTTATAATGTTATTGTCACTGCCCATGCCTTCGTAATAATTTTCTTTATAGTAA

TACCAATTATGATTGGAGGCTTCGGAAACTGACTTGTCCCCCTAATAATCGGGGCACCAG

ACATGGCATTTCCCCGAATAAATAATATGAGCTTTTGATTACTTCCCCCCTCCTTTCTAC

TACTGCTCGCCTCATCTGGGGTCGAGGCAGGGGCAGGAACAGGATGAACTGTATATCCAC

CGCTAGCTGGAAACCTTGCACACGCCGGAGCTTCCGTAGATCTAACTATTTTTTCCCTTC

ATTTGGCGGGGGTATCATCTATTCTAGGAGCCATTAATTTTATTACAACTATTATTAACA

TGAAACCCCCAGCAATTTCACAGTACCAAACACCTCTATTTGTGTGAGCTGTATTAATTA

CAGCTGTACTTCTACTGCTATCCCTACCCGTACTAGCCGCTGGCATTACAATACTTCTAA

CAGATCGAAATTTAAACACTACATTCTTCGACCCC

11_Pangasius_icaria_sp_nov.

CAGCCCTTAGCCTGCTAATTCGGGCTGAGCTAGCCCAGCCCGGCGCCCTTCTGGGTGATG

ACCAAATTTATAATGTTATTGTCACTGCCCATGCCTTCGTAATAATTTTCTTTATAGTAA

TACCAATTATGATTGGAGGCTTCGGAAACTGACTTGTCCCCCTAATAATCGGGGCACCAG

ACATGGCATTTCCCCGAATAAATAATATGAGCTTTTGATTACTTCCCCCCTCCTTTCTAC

TACTGCTCGCCTCATCTGGGGTCGAGGCAGGGGCAGGAACAGGATGAACTGTATATCCAC

CGCTAGCTGGAAACCTTGCACACGCCGGAGCTTCCGTAGATCTAACTATTTTTTCCCTTC

ATTTGGCGGGGGTATCATCTATTCTAGGAGCCATTAATTTTATTACAACTATTATTAACA

TGAAACCCCCAGCAATTTCACAGTACCAAACACCTCTATTTGTGTGAGCTGTATTAATTA

CAGCTGTACTTCTACTGCTATCCCTACCCGTACTAGCCGCTGGCATTACAATACTTCTAA

CAGATCGAAATTTAAACACTACATTCTTCGACCCC

1_Pangasius_icaria_sp_nov.

CAGCCCTTAGCCTGCTAATTCGGGCTGAGCTAGCCCAGCCCGGCGCCCTTCTGGGTGATG

ACCAAATTTATAATGTTATTGTCACTGCCCATGCCTTCGTAATAATTTTCTTTATAGTAA

TACCAATTATGATTGGAGGCTTCGGAAACTGACTTGTCCCCCTAATAATCGGGGCACCAG

ACATGGCATTTCCCCGAATAAATAATATGAGCTTTTGATTACTTCCCCCCTCCTTTCTAC

TACTGCTCGCCTCATCTGGGGTCGAGGCAGGGGCAGGAACAGGATGAACTGTATATCCAC

CGCTAGCTGGAAACCTTGCACACGCCGGAGCTTCCGTAGATCTAACTATTTTTTCCCTTC

ATTTGGCGGGGGTATCATCTATTCTAGGAGCCATTAATTTTATTACAACTATTATTAACA

TGAAACCCCCAGCAATTTCACAGTACCAAACACCTCTATTTGTGTGAGCTGTATTAATTA

CAGCTGTACTTCTACTGCTATCCCTACCCGTACTAGCCGCTGGCATTACAATACTTCTAA

CAGATCGAAATTTAAACACTACATTCTTCGACCCC

2_Pangasius_icaria_sp_nov.

CAGCCCTTAGCCTGCTAATTCGGGCTGAGCTAGCCCAGCCCGGCGCCCTTCTGGGTGATG

ACCAAATTTATAATGTTATTGTCACTGCCCATGCCTTCGTAATAATTTTCTTTATAGTAA

TACCAATTATGATTGGAGGCTTCGGAAACTGACTTGTCCCCCTAATAATCGGGGCACCAG

ACATGGCATTTCCCCGAATAAATAATATGAGCTTTTGATTACTTCCCCCCTCCTTTCTAC

TACTGCTCGCCTCATCTGGGGTCGAGGCAGGGGCAGGAACAGGATGAACTGTATATCCAC

CGCTAGCTGGAAACCTTGCACACGCCGGAGCTTCCGTAGATCTAACTATTTTTTCCCTTC

ATTTGGCAGGGGTATCATCTATTCTAGGAGCCATTAATTTTATTACAACTATTATTAACA

TGAAACCCCCAGCAATTTCACAGTACCAAACACCTCTATTTGTGTGAGCTGTATTAATTA

CAGCTGTACTTCTACTGCTATCCCTACCCGTACTAGCCGCTGGCATTACAATACTTCTAA

CAGATCGAAATTTAAACACTACATTCTTCGACCCC

3_Pangasius_icaria_sp_nov.

CAGCCCTTAGCCTGCTAATTCGGGCTGAGCTAGCCCAGCCCGGCGCCCTTCTGGGTGATG

ACCAAATTTATAATGTTATTGTCACTGCCCATGCCTTCGTAATAATTTTCTTTATAGTAA

TACCAATTATGATTGGAGGCTTCGGAAACTGACTTGTCCCCCTAATAATCGGGGCACCAG

ACATGGCATTTCCCCGAATAAATAATATGAGCTTTTGATTACTTCCCCCCTCCTTTCTAC

TACTGCTCGCCTCATCTGGGGTCGAGGCAGGGGCAGGAACAGGATGAACTGTATATCCAC

CGCTAGCTGGAAACCTTGCACACGCCGGAGCTTCCGTAGATCTAACTATTTTTTCCCTTC

ATTTGGCGGGGGTATCATCTATTCTAGGAGCCATTAATTTTATTACAACTATTATTAACA

TGAAACCCCCAGCAATTTCACAGTACCAAACACCTCTATTTGTGTGAGCTGTATTAATTA

CAGCTGTACTTCTACTGCTATCCCTACCCGTACTAGCCGCTGGCATTACAATACTTCTAA

CAGATCGAAATTTAAACACTACATTCTTCGACCCC

4_Pangasius_icaria_sp_nov.

CAGCCCTTAGCCTGCTAATTCGGGCTGAGCTAGCCCAGCCCGGCGCCCTTCTGGGTGATG

ACCAAATTTATAATGTTATTGTCACTGCCCATGCCTTCGTAATAATTTTCTTTATAGTAA

TACCAATTATGATTGGAGGCTTCGGAAACTGACTTGTCCCCCTAATAATCGGGGCACCAG

ACATGGCATTTCCCCGAATAAATAATATGAGCTTTTGATTACTTCCCCCCTCCTTTCTAC

TACTGCTCGCCTCATCTGGGGTCGAGGCAGGGGCAGGAACAGGATGAACTGTATATCCAC

CGCTAGCTGGAAACCTTGCACACGCCGGAGCTTCCGTAGATCTAACTATTTTTTCCCTTC

ATTTGGCGGGGGTATCATCTATTCTAGGAGCCATTAATTTTATTACAACTATTATTAACA

TGAAACCCCCAGCAATTTCACAGTACCAAACACCTCTATTTGTGTGAGCTGTATTAATTA

CAGCTGTACTTCTACTGCTATCCCTACCCGTACTAGCCGCTGGCATTACAATACTTCTAA

CAGATCGAAATTTAAACACTACATTCTTCGACCCC

5_Pangasius_icaria_sp_nov.

CAGCCCTTAGCCTGCTAATTCGGGCTGAGCTAGCCCAGCCCGGCGCCCTTCTGGGTGATG

ACCAAATTTATAATGTTATTGTCACTGCCCATGCCTTCGTAATAATTTTCTTTATAGTAA

TACCAATTATGATTGGAGGCTTCGGAAACTGACTTGTCCCCCTAATAATCGGGGCACCAG

ACATGGCATTTCCCCGAATAAATAATATGAGCTTTTGATTACTTCCCCCCTCCTTTCTAC

TACTGCTCGCCTCATCTGGGGTCGAGGCAGGGGCAGGAACAGGATGAACTGTATATCCAC

CGCTAGCTGGAAACCTTGCACACGCCGGAGCTTCCGTAGATCTAACTATTTTTTCCCTTC

ATTTGGCGGGGGTATCATCTATTCTAGGAGCCATTAATTTTATTACAACTATTATTAACA

TGAAACCCCCAGCAATTTCACAGTACCAAACACCTCTATTTGTGTGAGCTGTATTAATTA

CAGCTGTACTTCTACTGCTATCCCTACCCGTACTAGCCGCTGGCATTACAATACTTCTAA

CAGATCGAAATTTAAACACTACATTCTTCGACCCC

6_Pangasius_icaria_sp_nov.

CAGCCCTTAGCCTGCTAATTCGGGCTGAGCTAGCCCAGCCCGGCGCCCTTCTGGGTGATG

ACCAAATTTATAATGTTATTGTCACTGCCCATGCCTTCGTAATAATTTTCTTTATAGTAA

TACCAATTATGATTGGAGGCTTCGGAAACTGACTTGTCCCCCTAATAATCGGGGCACCAG

ACATGGCATTTCCCCGAATAAATAATATGAGCTTTTGATTACTTCCCCCCTCCTTTCTAC

TACTGCTCGCCTCATCTGGGGTCGAGGCAGGGGCAGGAACAGGATGAACTGTATATCCAC

CGCTAGCTGGAAACCTTGCACACGCCGGAGCTTCCGTAGATCTAACTATTTTTTCCCTTC

ATTTGGCGGGGGTATCATCTATTCTAGGAGCCATTAATTTTATTACAACTATTATTAACA

TGAAACCCCCAGCAATTTCACAGTACCAAACACCTCTATTTGTGTGAGCTGTATTAATTA

CAGCTGTACTTCTACTGCTATCCCTACCCGTACTAGCCGCTGGCATTACAATACTTCTAA

CAGATCGAAATTTAAACACTACATTCTTCGACCCC

7_Pangasius_icaria_sp_nov.

CAGCCCTTAGCCTGCTAATTCGGGCTGAGCTAGCCCAGCCCGGCGCCCTTCTGGGTGATG

ACCAAATTTATAATGTTATTGTCACTGCCCATGCCTTCGTAATAATTTTCTTTATAGTAA

TACCAATTATGATTGGAGGCTTCGGAAACTGACTTGTCCCCCTAATAATCGGGGCACCAG

ACATGGCATTTCCCCGAATAAATAATATGAGCTTTTGATTACTTCCCCCCTCCTTTCTAC

TACTGCTCGCCTCATCTGGGGTCGAGGCAGGGGCAGGAACAGGATGAACTGTATATCCAC

CGCTAGCTGGAAACCTTGCACACGCCGGAGCTTCCGTAGATCTAACTATTTTTTCCCTTC

ATTTGGCGGGGGTATCATCTATTCTAGGAGCCATTAATTTTATTACAACTATTATTAACA

TGAAACCCCCAGCAATTTCACAGTACCAAACACCTCTATTTGTGTGAGCTGTATTAATTA

CAGCTGTACTTCTACTGCTATCCCTACCCGTACTAGCCGCTGGCATTACAATACTTCTAA

CAGATCGAAATTTAAACACTACATTCTTCGACCCC

8_Pangasius_icaria_sp_nov.

CAGCCCTTAGCCTGCTAATTCGGGCTGAGCTAGCCCAGCCCGGCGCCCTTCTGGGTGATG

ACCAAATTTATAATGTTATTGTCACTGCCCATGCCTTCGTAATAATTTTCTTTATAGTAA

TACCAATTATGATTGGAGGCTTCGGAAACTGACTTGTCCCCCTAATAATCGGGGCACCAG

ACATGGCATTTCCCCGAATAAATAATATGAGCTTTTGATTACTTCCCCCCTCCTTTCTAC

TACTGCTCGCCTCATCTGGGGTCGAGGCAGGGGCAGGAACAGGATGAACTGTATATCCAC

CGCTAGCTGGAAACCTTGCACACGCCGGAGCTTCCGTAGATCTAACTATTTTTTCCCTTC

ATTTGGCGGGGGTATCATCTATTCTAGGAGCCATTAATTTTATTACAACTATTATTAACA

TGAAACCCCCAGCAATTTCACAGTACCAAACACCTCTATTTGTGTGAGCTGTATTAATTA

CAGCTGTACTTCTACTGCTATCCCTACCCGTACTAGCCGCTGGCATTACAATACTTCTAA

CAGATCGAAATTTAAACACTACATTCTTCGACCCC

9_Pangasius_icaria_sp_nov.

CAGCCCTTAGCCTGCTAATTCGGGCTGAGCTAGCCCAGCCCGGCGCCCTTCTGGGTGATG

ACCAAATTTATAATGTTATTGTCACTGCCCATGCCTTCGTAATAATTTTCTTTATAGTAA

TACCAATTATGATTGGAGGCTTCGGAAACTGACTTGTCCCCCTAATAATCGGGGCACCAG

ACATGGCATTTCCCCGAATAAATAATATGAGCTTTTGATTACTTCCCCCCTCCTTTCTAC

TACTGCTCGCCTCATCTGGGGTCGAGGCAGGGGCAGGAACAGGATGAACTGTATATCCAC

CGCTAGCTGGAAACCTTGCACACGCCGGAGCTTCCGTAGATCTAACTATTTTTTCCCTTC

ATTTGGCGGGGGTATCATCTATTCTAGGAGCCATTAATTTTATTACAACTATTATTAACA

TGAAACCCCCAGCAATTTCACAGTACCAAACACCTCTATTTGTGTGAGCTGTATTAATTA

CAGCTGTACTTCTACTGCTATCCCTACCCGTACTAGCCGCTGGCATTACAATACTTCTAA

CAGATCGAAATTTAAACACTACATTCTTCGACCCC
