# Supplementary material for: Description of a new Pangasius (Valenciennes, 1840) species, from the Cauvery River extends distribution range of the genus up to South Western Ghats in peninsular India
Source: PeerJ. 2022 Nov 8;10:e14258. doi: 10.7717/peerj.14258 (PMC9651045; doi:10.7717/peerj.14258)
Supplement: Supplemental Information 4 [file peerj-10-14258-s004.docx]

**Supplementary Table S2a: Intraspecies genetic distance in individuals of *P. Icaria***

|  | *10- P. Icaria sp nov* | *11- P. Icaria sp nov* | *1-P. Icaria sp nov* | *2-P. Icaria sp nov* | *3-P. Icaria sp nov* | *4-P. Icaria sp nov* | *5-P. Icaria sp nov* | *6-P. Icaria sp nov* | *7-P. Icaria sp nov* | *8- P. Icaria sp nov* | *9- P. Icaria sp nov* |
| --- | --- | --- | --- | --- | --- | --- | --- | --- | --- | --- | --- |
| *10-P. icaria-sp nov* |  |  |  |  |  |  |  |  |  |  |  |
| *11-P. icaria-sp nov* | 0.00000 |  |  |  |  |  |  |  |  |  |  |
| *1-P. icaria-sp nov* | 0.00000 | 0.00000 |  |  |  |  |  |  |  |  |  |
| *2-P. icaria-sp nov* | 0.00175 | 0.00175 | 0.00175 |  |  |  |  |  |  |  |  |
| *3-P. icaria-sp nov* | 0.00000 | 0.00000 | 0.00000 | 0.00175 |  |  |  |  |  |  |  |
| *4-P. icaria-sp nov* | 0.00000 | 0.00000 | 0.00000 | 0.00175 | 0.00000 |  |  |  |  |  |  |
| *5-P. icaria-sp nov* | 0.00000 | 0.00000 | 0.00000 | 0.00175 | 0.00000 | 0.00000 |  |  |  |  |  |
| *6-P. icaria-sp nov* | 0.00000 | 0.00000 | 0.00000 | 0.00175 | 0.00000 | 0.00000 | 0.00000 |  |  |  |  |
| *7-P. icaria-sp nov* | 0.00000 | 0.00000 | 0.00000 | 0.00175 | 0.00000 | 0.00000 | 0.00000 | 0.00000 |  |  |  |
| *8-P. icaria-sp nov* | 0.00000 | 0.00000 | 0.00000 | 0.00175 | 0.00000 | 0.00000 | 0.00000 | 0.00000 | 0.00000 |  |  |
| *9-P. icaria-sp nov* | 0.00000 | 0.00000 | 0.00000 | 0.00175 | 0.00000 | 0.00000 | 0.00000 | 0.00000 | 0.00000 | 0.00000 |  |

|  | *P._hypophthalmus* | *P._pangasius* | *P._icaria* | *P._silasi* | *P._mekongensis* | *P._larnaudii* | *P._boourti* | *P._sanitwongsei* | *P._nasutus* | *P._krempfi* | *P._macronema* | *P._conchophilus* | *P._elongatus* | *P._djambal* |
| --- | --- | --- | --- | --- | --- | --- | --- | --- | --- | --- | --- | --- | --- | --- |
| *P._hypophthalmus* |  |  |  |  |  |  |  |  |  |  |  |  |  |  |
| *P._pangasius* | 0.1546 |  |  |  |  |  |  |  |  |  |  |  |  |  |
| *P._icaria* | 0.1437 | 0.0477 |  |  |  |  |  |  |  |  |  |  |  |  |
| *P._silasi* | 0.1487 | 0.0489 | 0.0295 |  |  |  |  |  |  |  |  |  |  |  |
| *P._mekongensis* | 0.1454 | 0.0564 | 0.0722 | 0.0625 |  |  |  |  |  |  |  |  |  |  |
| *P._larnaudii* | 0.1436 | 0.0938 | 0.0928 | 0.0968 | 0.0912 |  |  |  |  |  |  |  |  |  |
| *P._boourti* | 0.1285 | 0.0944 | 0.0901 | 0.0966 | 0.0969 | 0.1078 |  |  |  |  |  |  |  |  |
| *P._sanitwongsei* | 0.1548 | 0.1027 | 0.1000 | 0.1197 | 0.1187 | 0.1183 | 0.0846 |  |  |  |  |  |  |  |
| *P._nasutus* | 0.1259 | 0.0849 | 0.0813 | 0.0875 | 0.0774 | 0.0852 | 0.0734 | 0.0794 |  |  |  |  |  |  |
| *P._krempfi* | 0.1312 | 0.1143 | 0.1111 | 0.1080 | 0.1070 | 0.1143 | 0.1051 | 0.1092 | 0.1083 |  |  |  |  |  |
| *P._macronema* | 0.1572 | 0.1366 | 0.1292 | 0.1397 | 0.1331 | 0.1166 | 0.1012 | 0.1201 | 0.0879 | 0.1232 |  |  |  |  |
| *P._conchophilus* | 0.1236 | 0.0838 | 0.0810 | 0.0874 | 0.0747 | 0.0815 | 0.0706 | 0.0744 | 0.0034 | 0.1060 | 0.0824 |  |  |  |
| *P._elongatus* | 0.1515 | 0.1311 | 0.1265 | 0.1397 | 0.1331 | 0.1115 | 0.0963 | 0.1148 | 0.0786 | 0.1285 | 0.0098 | 0.0730 |  |  |
| *P._djambal* | 0.1231 | 0.0978 | 0.0886 | 0.1001 | 0.0992 | 0.1063 | 0.0022 | 0.0868 | 0.0709 | 0.1024 | 0.0997 | 0.0679 | 0.0949 |  |
|  |  |  |  |  |  |  |  |  |  |  |  |  |  |  |

**Supplementary Table S2b: Interspecies genetic distance of *P. Icaria* with other congeners**
